# Osseointegration by bone morphogenetic protein-2 and transforming growth factor beta2 coated titanium implants in femora of New Zealand white rabbits

**DOI:** 10.4103/0019-5413.73659

**Published:** 2011

**Authors:** Fritz Thorey, Henning Menzel, Corinna Lorenz, Gerhard Gross, Andrea Hoffmann, Henning Windhagen

**Affiliations:** Department of Orthopaedic Surgery, Hannover Medical School, Hannover, Germany; 1 Institute for Technical Chemistry, Braunschweig University of Technology, Braunschweig, Germany; 2 Helmholtz Centre for Infection Research, Department of Gene Regulation and Differentiation, Braunschweig, Germany; 3 Department of Trauma Surgery, Hannover Medical School, Hannover, Germany

**Keywords:** BMP-2, TGF-β2, osseointegration

## Abstract

**Background:**

Intramembranous bone formation is essential in uncemented joint replacement to provide a mechanical anchorage of the implant. Since the discovery of bone morphogenic proteins (BMPs) by Urist in 1965, many studies have been conducted to show the influence of growth factors on implant ingrowth. In this study, the influence of bone morphogenetic protein-2 (rhBMP-2) and transforming growth factor β2 (TGF-β2) on implant osseointegration was investigated.

**Materials and Methods:**

Thirty-two titanium cylinders were implanted into the femoral condyles of both hind legs of New Zealand White Rabbits. Four experimental groups were investigated: controls without coating, a macromolecular copolymer + covalently bound BMP-2, adsorbed BMP-2, and absorbed BMP-2+TGF-β2. All samples were analyzed by *ex vivo* high-resolution micro-computed-tomography after 28 days of healing. Bone volume per total volume (BV/TV) was recorded around each implant. Afterward, all samples were biomechanically tested in a pull-out setup.

**Results:**

The highest BV/TV ratio was seen in the BMP-2 group, followed by the BMP-2+TGF-β2 group in high-resolution micro-computed-tomography. These groups were significantly different compared to the control group (*P* < 0.05). Copolymer+BMP-2 showed no significant difference in comparison to controls. In the pull-out setup, all groups showed higher fixation strength compared to the control group; these differences were not significant.

**Conclusions:**

No differences between BMP-2 alone and a combination of BMP-2+TGF-β2 could be seen in the present study. However, the results of this study confirm the results of other studies that a coating with growth factors is able to enhance bone implant ingrowth. This may be of importance in defect situations during revision surgery to support the implant ingrowth and implant anchorage.

## INTRODUCTION

Intramembranous bone formation is essential in uncemented joint replacement to provide a mechanical anchorage of the implant.^1^ Analyses in revision surgery have shown wide variations of implant osseointegration.^1^,^2^ Therefore, other supportive methods to increase bone ingrowth were investigated. Especially in maxillofacial surgery, many studies have been conducted to improve the quality and quantity of implant osseointegration; e.g., improvement of implant biocompatibility and modification of surface characteristics,^3^ grafting of autograft-bone or allograft-bone,^4^,^5^ and modified surgical techniques.^6^–^8^ Since the discovery of Bone Morphogenetic Proteins (BMPs) by Urist (1965) and their molecular cloning and characterization, they have already found their way into clinical practice for specific indications.^9^–^14^ BMP-2 and transforming growth factor β (TGF-β) have been shown to stimulate bone ingrowth, gap healing, and implant fixation in several animal studies.^15^–^26^ Other animal studies have demonstrated that titanium implants are a sufficient carrier for these growth factors.^27^–^32^

Thus, the aim of this study was to investigate the influence of bone morphogenetic protein-2 (rhBMP-2) and TGF-b2 on implant osseointegration using high-resolution micro-computed-tomography and biomechanical methods in an animal model of New Zealand White Rabbits.

## MATERIALS AND METHODS

Eight mature New Zealand White Rabbits were used. They were housed in standard laboratory conditions. All animals were fed with autoclaved water and food. All animals were investigated preoperatively by a veterinarian. This included a general health check and an examination of parasites. The study was approved by the Institutional Animal Care and Use Committee, Germany.

**Figure 1:**
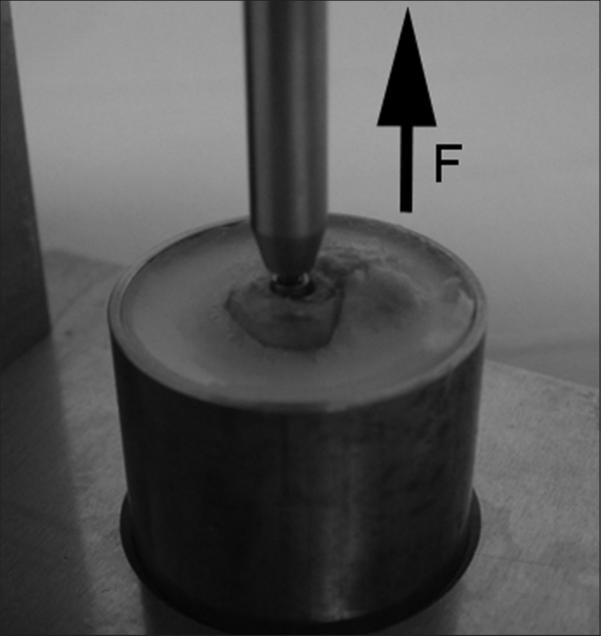
Embedded sample in the MTS Mini Bionix 858 Test Star (MTS Systems Corporation, Minneapolis, USA) for the pull-out test

Thirty-two implants were designed as titanium cylinders (Ti90Al6V4, Ø 3 mm × 3 mm, average roughness 95 nm, Goodfellow GmbH, Germany) with an innerthread for the biomechanical test. The innerthread was designed to screw a special manufactured stem in for the pull-out test [Figure 1]. They were divided into four groups according to four different surface chemistries to be tested. Eight control cylinders (group 1) consisted of nonmodified titanium. Eight titanium cylinders were coated with a copolymer as an anchor for rhBMP-2 (group 2): as a copolymer, poly-vinylbenzylphosphonate-co-glycidylmethacrylate (p-VBP-co-GMA) was used to provide a special anchor on the surface of the implant. The coating was done by a dip-coating technique. Afterward, the BMP-2 was bound covalently via the epoxy groups of the polymer and the unbound BMP-2 was removed by a special washing process. The amount of BMP-2 on the surface of each cylinder coated with copolymer was estimated to be > 100 ng/cm2 by ELISA depending on the layer thickness of the polymer (group 3). Group 3 was made up of eight cylinders which were exposed to 50 *µ*l of BMP2-solution (250 *µ*g/ml), which was allowed to evaporate overnight under sterile conditions. This serves as a positive control of nonspecifically absorbed BMP2. In group 4, the use of a solution of BMP2 plus TGF-b2 (12.5 ng/*µ*l) for nonspecific coating of the cylinders resulted in 12.5 *µ*g BMP2 plus 625 ng TGF-b2 per cylinder. All four different groups of cylinders were implanted in each animal.

### Operative procedure

The animals were preanesthetized with 25 mg/kg ketamin and 5 mg midazolam intramuscularly. A sterile catheter was placed and the anesthesia was started using propofol. After intubation the anesthesia was maintained with isofluran and a ringer-solution. A broad-spectrum antibiotic (tardomyocel comp. III) and analgesic (buphrenorphin) was applied.

**Figure 2:**
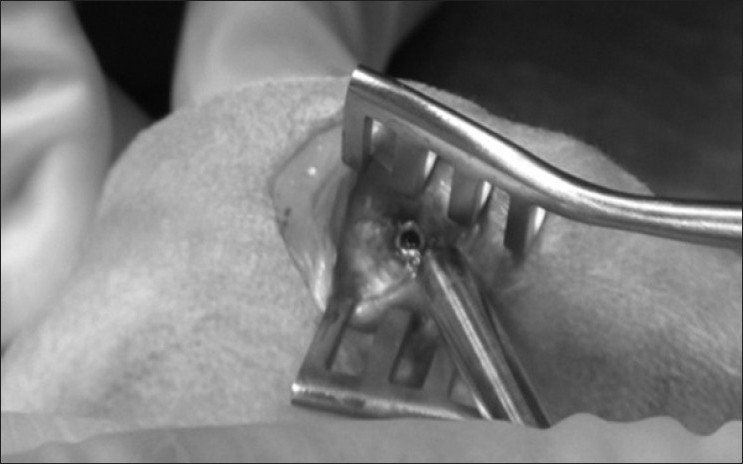
Intraoperative photograph of an implant in the medial condyle of a New Zealand White Rabbit

Surgery was done by one surgeon under the same condition. The animals were placed in supine position on the operating table. After disinfection of both hind legs and sterile coverage of the animal, a small skin incision was made with a scalpel above the patella tendon. The incision was moved easily medially or laterally to perform the subcutaneous incision directly to the bone. This technique was used to minimize infection. The second step was to display the medial condyle of the femur. Using a wound spreader, the periosteum and bone could be easily shown. A hand drill (3 mm diameter) was used to drill a hole to fit the implant [Figure 2]. The implant was placed in the cancellous bone using a special inserter. Afterward, the skin was moved for the implantation on the lateral condyle. The same procedure was performed on the other leg. To minimize a systemic failure of different conditions of the cancellous bone in the medial and lateral condyle, the positioning of all cylinders alternated between each animal. The skin was closed with absorbable sutures (Vicryl, Ethicon/Johnson and Johnson, Germany) for healing by primary intention. Three days postsurgery, all animals received 4 mg/kg Carprofen subcutaneously.

After 28 days of surgery, all animals were euthanized to analyze the early implant osseointegration.^33^ They were sedated with 10 mg/kg ketamin and euthanized with an overdose pentobarbital (Eutha 77). Both hind legs were extracted, the femoral condyle removed and stored in formalin.

### High-resolution micro-computed-tomography

**Figure 3:**
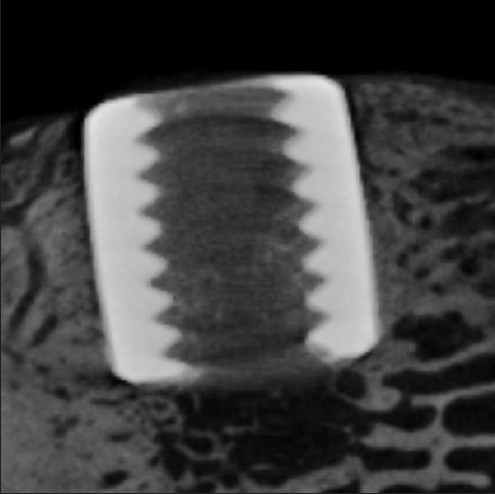
High-resolution micro-computed-tomography of an implanted titanium cylinder. The inner thread was used for the implantation and the pull-out test

All samples were analyzed in an *ex vivo* high-resolution micro-computed-tomography apparatus (Centre for Synchrotron Radiation, University of Technology, Dortmund, Germany) using synchrotron radiation [Figure 3]. With special adapted software, the region of interest around each titanium cylinder (300 *µ*m) was analyzed. This nondestructive method enables a fast, three-dimensional, and quantitative measurement of the bone tissue around implants.^34^ The measurement units were 1 Voxel (35 *µ*m). After a defined segmentation process, a global threshold was defined as barrier between implant and bone and also between bone and soft-tissue. The analysis produces a quotient of bone volume to total volume (BV/TV). These data can be seen as bone ingrowth of implants when compared to the cylinder of each other group.

### Mechanical testing

All samples were embedded in Technovit 4004 (Heraeus Kulzer GmbH, Wehrheim, Germany) and fixed in a special block. The fixation strength was measured by a MTS Mini Bionix 858 Test Star (MTS Systems Corporation, Minneapolis, USA). The mechanical testing was accomplished at a rate of 0.5 mm/sec with a longitudinal force direction to the implant axis. All data were recorded by the Test Star II software for statistical analysis.

### Statistics

Mean values and standard deviation were analyzed for all groups. Furthermore, the independent-samples *t*-test was used to analyze the differences in fixation strength and BV/TV between all groups. *P* < 0.05 was considered statistically significant.

## RESULTS

No complications (e.g., infection, fracture) were found during the examination period of 28 days. All extracted samples could be used for the high-resolution micro-computed-tomography and biomechanical pull-out test.

### High-resolution micro-computed-tomography

A region of interest was defined around each cylinder with 300 *µ*m width. In this three-dimensional area, the software was able to differentiate between bone and nonbone (soft-tissue). To homogenize all received data independently of their implant location and bone stock quality in each animal, the control cylinder was assumed to be 100% bone ingrowth and the coated cylinders were compared relatively to the control in percentage. This enabled a comparison of all animals, independent of their individual cancellous bone stock in the condyle.

The highest ingrowth of implant was seen in the BMP-2 group (115.4 ± 7.5%), followed by the BMP-2 + TGF-b2 group (113.5 ± 7.2%). The copolymer + BMP-2 group was found to be 103.0 ± 2.3%. There were no significant differences between groups 2, 3, and 4.

**Figure 4:**
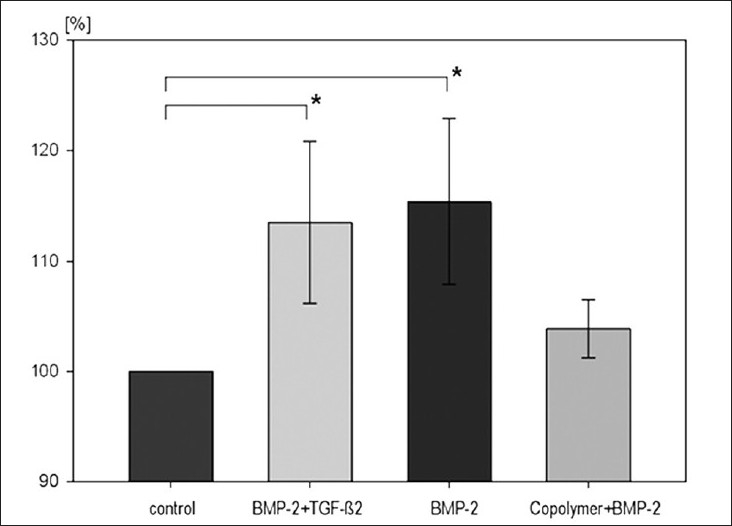
Bar diagram showing osseointegration of implants. BV/ TV(%) with standard error of all groups (*significant difference to control group; *P* < 0.05)

BMP-2 group (*P* < 0.05) and BMP-2 + TGF-b2 group (*P* < 0.05) were significantly different compared to the control group. The copolymer + BMP-2 group was not significantly different compared to the control group (P=0.17) [Figure 4].

### Mechanical testing

The fixation strength was defined as the point of failure during the biomechanical pull-out test when the implant can be removed. To homogenize all received data independently of their implant location and bone stock quality of each animal, the control cylinder was assumed to be 100% fixation strength and the coated cylinders were compared relatively to the control in percentage. This enabled a comparison of all animals, independently of their individual cancellous bone stock in the condyle.

**Figure 5:**
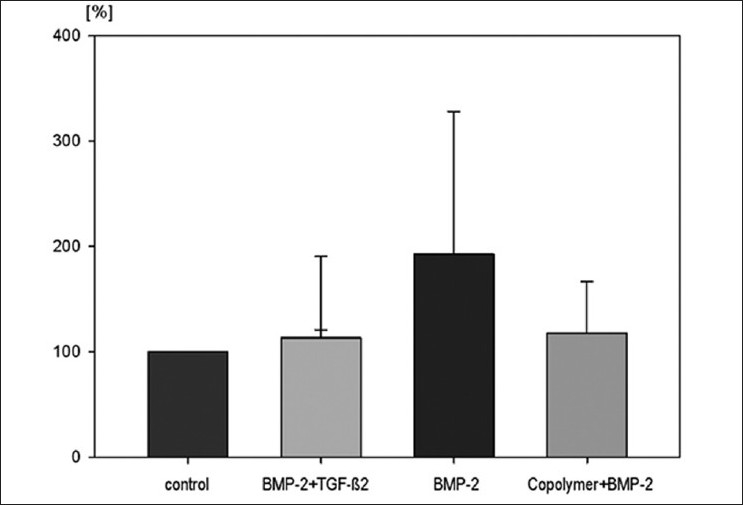
Bar diagram showing maximum pull-out strength (N) with standard error of all groups. No significant differences between all groups

The highest pull-out strength was found in the BMP-2 group (192.5 ± 135.4%). The pull-out strength of the copolymer + BMP-2 group was 117.6 ± 49.1% and of the BMP-2 + TGF-b2 group was 113.0 ± 77.4%. There was no significant difference between control group and each other group (*P* > 0.5), but a trend of increased implant ingrowth especially in the BMP-2 group [Figure 5].

## DISCUSSION

In this study, the effect of different titanium implant coatings on bone ingrowth was analyzed. Titanium implants without any surface coating served as control. All implants were implanted in the medial and lateral condyle of both femora in New Zealand White Rabbits.

In high-resolution micro-computed-tomography, we found a significant difference of the BMP-2 group (*P* < 0.05) and BMP-2 + TGF-β2 group (*P* < 0.05), compared to the control group. The combination of copolymer as an anchor for covalent binding of BMP-2 on the titanium surface showed an increase in implant ingrowth, but the difference was not significant. The pull-out test showed the same distribution, but no significant differences between coated implants and the control group.

Mechanical fixation of implants is an important consideration because this influences the response of the bone toward the implant and its. Three types of mechanical tests are commonly used: torque, push-in, and pull-out. In this study, pull-out was used because of the thinness of rabbit bone.

The ingrowth of implants in bone depends on bone remodeling and modeling by existing osteoblasts and osteoclasts. This leads to osseointegration of the implant. Differentiation of various cell types (undifferentiated mesenchymal stem cells, osteoprogenitor cells, monocytes) into osteoblasts and osteoclasts leads to osteoid production and mineralized bone under the influence of locally acting growth factors. BMPs, as a member of the TGF-β superfamily, have a variety of functions in the development and reparation of bone tissue. Several studies have shown the induction of osteoblast proliferation, differentiation, and influence on bone formation.^35^,^36^ Among more than 20 described isoforms, BMP-2, BMP-4, and BMP-7 play an important role in the bony skeletal system. Especially BMP-2 has been shown to have an important function in defect repair and implant osseointegration in maxillofacial surgery.^37^–^40^ Depending on their concentration gradient, BMPs can attract various types of cells, acting as differentiation, chemotactic or mitogenic agents.^41^–^43^ In combination with BMP-2, TGF-b2 has been demonstrated to cause an increase in bone ingrowth in a canine study after 4 weeks on implants coated additionally with hydroxyapatite-tricalcium phosphate.^44^ The combination of both growth factors has shown a synergistic effect on implant ingrowth through related but separate signal transduction pathways; TGF-β with control of osteoprogenitor cell proliferation, BMPs with more important influence in osteoblasts differentiation.^45^,^46^

In the present study, the BMP-2 + TGF-β2 group showed a significant increase in high-resolution micro-computed-tomography but a nonsignificant increase in the mechanical testing. However, we have not found the clear superiority of BMP-2 + TGF-β2 compared to BMP-2 coating of implants as described in other studies.^44^ A possible explanation may be the lower concentration of growth factors in the present study. Sumner *et al*. demonstrated that 12 *µ*g TGF-β2 and 25 *µ*g BMP-2 is the optimum dose.^44^ Additionally they used a hydroxyapatite-tricalcium phosphate coating of each implant. In our study, we used 625 ng TGF-β2 and 12.5 *µ*g BMP2 to simulate a more physiological concentration of both growth factors. In the copolymer group, BMP-2 was linked covalently to the copolymer via a limited number of binding sites for the growth factor. This led to a smaller amount of BMP-2 immobilized on the implant surface that was reflected by a minimal increase of bone ingrowth compared to the BMP-2 group and BMP-2 + TGF-b2 group.

A limitation of the present study may be the lower concentration of growth factors on the implant surface compared to other studies that makes an interpretation of our results in comparison to other studies more demanding. Furthermore, the small number of animals may lead to failures in statistics and might change to significant level with a higher amount of animals. However, this study does present an increase in implant ingrowth in all three groups compared to the control group, which reflects the potency of growth factors during implant ingrowth.

In future, one has to consider the use of growth factors especially in revision total hip arthroplasty with great loss of bone stock, which can possibly replace or be added to bone grafts that are commonly used in uncemented revision cases.^47^ This may support surgeons in demanding and challenging situations. However, further studies with larger bone defects around implants should be conducted to transfer the results of small animal studies to large animals to define special doses of growth factors for individual defect and revision cases.
